# Atmospheric Air Pollution by Stationary Sources in Ulan-Ude (Buryatia, Russia) and Its Impact on Public Health

**DOI:** 10.3390/ijerph192416385

**Published:** 2022-12-07

**Authors:** Bair O. Gomboev, Irina K. Dambueva, Sergey S. Khankhareev, Valentin S. Batomunkuev, Natalya R. Zangeeva, Vitaly E. Tsydypov, Bayanzhargal B. Sharaldaev, Aldar G. Badmaev, Daba Ts.-D. Zhamyanov, Elena E. Bagaeva, Ekaterina V. Madeeva, Marina A. Motoshkina, Valentina G. Ayusheeva, Tumun Sh. Rygzynov, Aryuna B. Tsybikova, Alexander A. Ayurzhanaev, Bator V. Sodnomov, Zorikto E. Banzaraktcaev, Aleksei V. Alekseev, Aryuna B. Lygdenova, Beligma S. Norboeva

**Affiliations:** 1 Baikal Institute of Nature Management SB RAS, 670047 Ulan-Ude, Russia; 2 Department of Geography and Geoecology Chair, Faculty of Biology, Geography and Land Management, Banzarov Buryat State University, 670000 Ulan-Ude, Russia; 3 Institute of Biological Problems of the North FEB RAS, 685000 Magadan, Russia; 4 Federal Service for Supervision of Consumer Rights Protection and Human Welfare in Buryatia (Rospotrebnadzor), 670045 Ulan-Ude, Russia

**Keywords:** atmospheric air pollutants, benzo(*a*)pyrene, public health risk, population morbidity

## Abstract

For the first time in the territory of the Russian Far East, a study related to the establishment of correlations between air quality and public health in Ulan-Ude (Buryatia, Russia) was carried out. This study is based on the analysis of official medical statistics on morbidity over several years, the data on the composition and volume of emissions of harmful substances into the air from various stationary sources, and laboratory measurements of air pollutants in different locations in Ulan-Ude. This study confirmed that the morbidity of the population in Ulan-Ude has been increasing every year and it is largely influenced by air pollutants, the main of which are benzo(*a*)pyrene, suspended solids, PM_2.5_, PM_10_, and nitrogen dioxide. It was found that the greatest contribution to the unfavorable environmental situation is made by three types of stationary sources: large heating networks, autonomous sources (enterprises and small businesses), and individual households. The main air pollutants whose concentrations exceed the limits are benzo(*a*)pyrene, formaldehyde, suspended particles PM_2.5_, PM_10_, and nitrogen dioxide. A comprehensive assessment of the content of various pollutants in the atmospheric air showed that levels of carcinogenic and non-carcinogenic risks to public health exceeded allowable levels. Priority pollutants in the atmosphere of Ulan-Ude whose concentrations create unacceptable levels of risk to public health are benzo(*a*)pyrene, suspended solids, nitrogen dioxide, PM_2.5_, PM_10_, formaldehyde, and black carbon. The levels of morbidity in Ulan-Ude were higher than the average for Buryatia by the main disease classes: respiratory organs—by 1.19 times, endocrine system—by 1.25 times, circulatory system—by 1.11 times, eye diseases—by 1.06 times, neoplasms—by 1.47 times, congenital anomalies, and deformations and chromosomal aberrations—by 1.63 times. There is an increase in the incidence of risk-related diseases of respiratory organs and the circulatory system. A strong correlation was found between this growth of morbidity and atmospheric air pollution in Ulan-Ude.

## 1. Introduction

Modern urbanization in the cities of Siberia and the Far East leads to increased emissions of pollutants into the air [1,2,3,4]. The expansion of local pollution hotspots is becoming a regional problem and the number of pollutants continues to increase. According to international standards, the main pollutants are: fine suspended particles PM_2.5_ and PM_10_, sulfur dioxide (SO_2_), nitrogen dioxide (NO_2_), carbon oxide (CO), and ozone (O_3_) [5,6,7]. These air pollutants affect not only the air quality but also public health. PM_10_ and PM_2.5_ contain inhalable particles that are so small that they can penetrate the thoracic region of the respiratory system and thereby cause respiratory and cardiovascular morbidity, increasing the risk of cardiopulmonary mortality [8]. When SO_2_, NO_2_, and O_3_ pollutants penetrate the respiratory system, they cause chronic bronchitis, asthma, heart ischemia, and lung cancer [9,10,11,12,13]. Urban air pollution has attracted a great deal of attention from both public groups and governments at all levels [14]. Solving this problem has become an important research task. Many studies deal with pollutant concentrations, temporal and seasonal distribution, the influence of transport, and the correlation between atmospheric and meteorological factors [15,16]. The relationship between the level of mortality in Russian regions from different causes and the level of atmospheric pollution has also been analyzed [17].

In 2021, the number of additional deaths from all causes related to atmospheric air pollution in residential areas was probabilistically amounted to 4.6 cases per 100 thousand people on average in Russia (or 0.31% of the actual mortality rate of the population of Russia). In the territory of 15 Russian regions in 2021, population mortality from malignant neoplasms was probabilistically associated with atmospheric air pollution, the number of additional cases was in the range from 0.1 to 63.7 cases per 100 thousand population. Average Russian levels were exceeded in the territories of nine regions in the range from 2.5 to 32.7 times. The highest levels were recorded in Zabaikalsky Krai, Kemerovo Oblast, Krasnoyarsk Krai, Buryatia, and Chelyabinsk Oblast (8.5–63.7 cases per 100 thousand population) [4,18]. 

In the settlements of Eastern Siberia, even in the absence of large industries with emission sources, the state of atmospheric air is unsatisfactory during the long heating period.

Ulan-Ude is the administrative center of the Republic of Buryatia; the city’s population is 436,400 people. Ulan-Ude is annually included in the Priority List of Russian cities with the highest level of air pollution according to the Federal Service for Hydrometeorology and Environmental Monitoring (Rosgidromet). The average annual concentrations of benzo(*a*)pyrene, suspended substances, PM_2.5_, PM_10_, and formaldehyde annually exceed the maximum allowable concentrations [17,19,20,21,22,23,24,25,26].

The greatest contribution to air pollution in Ulan-Ude is made by enterprises of the fuel and energy complex (two central heating and power plants in Ulan-Ude—CHPP-1 and CHPP-2, large centralized boilers of the Ulan-Ude energy complex, which are part of PAO “Territorial Generating Company No 14” and numerous small boilers), individual households, and motor transport [27,28,29,30,31,32,33,34].

Moreover, the climatic and topographic conditions (mountain and basin topography), which are very unfavorable for the dispersion of impurities, contribute to the accumulation of harmful substances in the surface layer of the atmosphere [24,35]. The territory of Ulan-Ude refers to a zone of high air pollution potential (hereinafter—APP) where meteorological conditions of pollutant dispersion in the atmosphere contribute to the transfer of harmful substances over considerable distances [36]. Atmospheric air pollution in Ulan-Ude has been worsening due to the expansion of household development in the suburban areas of Tarbagataisky, Ivolginsky, and Zaigraevsky districts. Over the past decade, the number of individual households with autonomous heating boilers and stoves has increased from 20,000 to 77,000, according to preliminary estimates. The Government of Buryatia, executive authorities, and municipalities receive complaints from residents about air pollution during the heating period.

The condition of atmospheric air is one of the priority environmental factors affecting public health. High levels of atmospheric air pollution can cause diseases of the respiratory organs, cardiovascular system, central nervous system, vision, blood, oncopathology, as well as developmental and immune system disorders [37].

In this paper we attempt to establish a correlation between the morbidity of the population of Ulan-Ude and atmospheric air pollution. This study aimed to establish the impact of atmospheric air pollution from stationary sources on the health of the population of Ulan-Ude.

The research objectives are as follows: (1) to analyze the data on the state of atmospheric air pollution in Ulan-Ude; (2) to analyze the main sources of atmospheric air pollution in Ulan-Ude as well as the additional research data on the use of fuel at autonomous heating sources, i.e., individual households and small boilers of enterprises; (3) to determine the levels of health risks for the population of Ulan-Ude when exposed to polluted atmospheric air; (4) to analyze the morbidity indicators of the population of Ulan-Ude and correlating them with the level of atmospheric air pollution.

## 2. Materials and Methods

In this study, we analyzed the composition and volume of emissions of pollutants into the atmospheric air by economic entities according to the methodology approved by Rosstat Order No. 661 dated 8 November 2018 “On Approval of the Methodology for Statistical Observation of Atmospheric air Protection” (form No. 2-TP “Information on atmospheric air protection”). We used data on the average annual concentrations of pollutants in the atmospheric air of Ulan-Ude that were obtained from meteorological stations that are operated by the Buryat Center for Hydrometeorology and Environmental Monitoring (hereinafter—the Buryat CHEM) for 2011–2020. We also analyzed data on atmospheric air pollution at monitoring sites in Ulan-Ude, measured by the experts of the Federal Service for Supervision of Consumer Rights Protection and Human Welfare in Buryatia (Rospotrebnadzor).

As part of this study, the concentrations of pollutants in air emissions from households were measured. The findings of this study are based not on calculation methods, but on actual data that were obtained for the first time for Ulan-Ude. These data largely clarify the composition and mass of pollutants that are emitted into the atmospheric air. Pollutant emissions from conventional fuel combustion were measured for 6 days. Measurements were taken during combustion and smoldering of fuel 3 times a day. 

The tests of atmospheric air in different periods of the year were carried out in an accredited laboratory of OOO “Occupational Safety and Health Certification Center” (Accreditation certificate RA.RU.21AI87, issued on 5 July 2016). A total of 324 air samples were taken during 54 surveys to measure the concentrations of pollutants.

All the measurements were made using methods that comply with Article 5 of the Federal Law No. 102 FZ “On Ensuring the Uniformity of Measurements”, using the following state standards GOST 17.2.4.07–90 [38], GOST 17.2.4.06–90 [39]; and official measurement methods: DKIN.413411.002 RE [40], GOST 33007–2014 [41], FR.1.31.2015.20718 (PNDF 13.1.76–15) [42,43]. Calibrated instruments included in the State Register of Measuring Instruments were used [44].

The gross pollutant emission for each type of heat source and fuel type was calculated using the following formula:

G*_i_* = *М_х_* ⋅ *t* ⋅ *P* ⋅ *N* ⋅ *k*, (1)

where *М_х_* is the pollutant emission rate (g/s), *t* is the total usage time of heat sources (h), *P* is the share of a particular type of heat source, *N* is the number of households, and *k* is the unit conversion factor (hours into seconds). 

The measured concentrations of pollutants were compared with the maximum allowable concentrations (MAC) approved by Decree of Chief State Sanitary Doctor of Russia No. 165 dated 22 December 2017 “On Approval of Hygienic Standards GN 2.1.6.3492–17 «Maximum allowable concentrations of pollutants in the air of urban and rural settlements»”.

We also geographically mapped the quantitative and qualitative composition of pollutant emissions and the concentration fields (pollution maps) provided by the municipal administration of Ulan-Ude. Analytical information from Rospotrebnadzor in Buryatia on the morbidity rate in Ulan-Ude in 2011–2020 was used. The source data on population morbidity were the statistical reporting forms No. 12 “Morbidity of patients residing in the service area of a medical organization”. Correlation analysis of population morbidity and atmospheric air pollution was performed in MS Excel.

## 3. Results

In this study, we analyzed data on atmospheric air pollution in Ulan–Ude for the ten–year period (2011–2020). The monitoring of atmospheric air in Ulan-Ude was carried out at three monitoring stations, operated by the Buryat CHEM, and seven monitoring sites, surveyed by the experts of Rospotrebnadzor (Table 1).

In 2020 (according to the Buryat CHEM data), the average annual concentrations in Ulan-Ude exceeded MACs for benzo(*a*)pyrene by 10.3 times, for suspended solids by 1.3 times, for nitrogen dioxide by 1.08 times, and for fine particulate matter PM_2.5_ and PM_10_ by 1.76 and 1.5 times, respectively. In 2020 relative to 2011, there was a 3.68-fold increase in the average annual concentrations of benzo(*a*)pyrene, 2.0-fold increase in sulfur dioxide, 1.25-fold increase in phenol (25.0%), 1.92-fold increase in nitrogen dioxide (91.67%), and a 1.08-fold increase in nitrogen oxide (7.5%). Also, in 2020 relative to 2017, there was a 1.31-fold (31.34%) increase in PM_2.5_ concentrations and a 1.25-fold (25.0%) increase in PM_10_. Over the entire study period (2011–2020), the average daily concentrations exceeded MACs for benzo(*a*)pyrene by 2.8–11.95 times, suspended solids by 1.3–1.88 times, formaldehyde by 1.1–2.3 times, nitrogen dioxide by 1.1–1.13 times, ozone by 1.07–1.53 times, PM_2.5_ by 1.34–1.76 times, and PM_10_ by 1.07–1.5 times.

The Air Pollution Index (API) is an integral indicator calculated on the basis of the average annual concentrations of pollutants, their MAC values, and the degree of their danger. To compare the pollution levels in various localities and years, the API_5_ index was calculated for the average annual concentrations of the five most important air pollutants (in accordance with the official regulatory document RD 52.04.667–2005, issued by Rosgidromet). An API in the range of 5–6 is regarded as elevated, 7–13—high, and ≥14—very high. In Ulan-Ude for the period 2011–2020, there was an increase in API values from 10.0 to 37.1 (Table 2). In 2011–2012, the degree of air pollution was rated as “high”, and from 2013 to 2020 it was “very high”.

**Figure 1:**
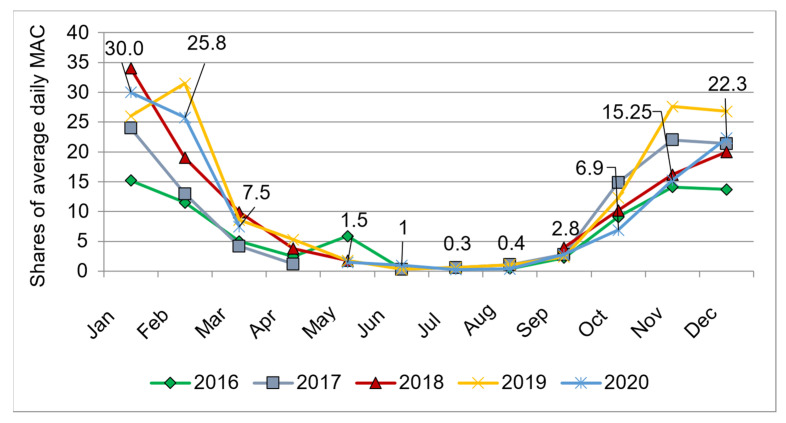
Annual dynamics of benzo(*a*)pyrene concentrations in the air of Ulan-Ude in 2016–2020, measured at the air monitoring stations (operated by the Buryat CHEM), shares of MAC.

The monitoring data since 2015 indicate that the most intense air pollution in Ulan-Ude is observed during the heating season, especially under adverse meteorological conditions (hereinafter referred to as AWC). According to the Buryat CHEM data, in January 2020, during the AWC period, benzo(*a*)pyrene concentrations exceeded the average daily MAC by 57.2 times (at the monitoring station on ulitsa Babushkina). In January 2020, the average daily concentrations of benzo(*a*)pyrene in Ulan-Ude exceeded MAC by 30.0 times, and in March—by 7.5 times (Figure 1).

According to the Directorate of Rospotrebnadzor in Buryatia, during the study period, the average daily concentrations of benzo(*a*)pyrene exceeded MAC in the 20th city blocks by 29.5 times, on ulitsa Tereshkovoi by 1.3 times, in Istok by 4.8 times, in Energetik by 8.5 times, in Gorky by 17.2 times, and on ulitsa Kluchevskaya by 33.6 times. According to long-term data, the highest level of air pollution in Ulan-Ude is registered annually in the cold period of the year due to increased emissions of pollutants from small boiler facilities and autonomous heating sources, including individual households, located around the city and in its central part. The ranking of monitoring sites in Ulan-Ude by the coefficient of total air pollution (K_sum_) for 2011–2021 showed that in the cold period of the year, pollution is rated as “very high” in Zarechny, Kirzavod, ulitsa Revolutsii 1905, and ulitsa Klyuchevskaya. During the warm period of the year, air pollution at all monitoring sites is rated as “moderate”.

The territory of Ulan-Ude refers to a zone of high air pollution potential where meteorological conditions of pollutant dispersion in the atmosphere contribute to the transfer of harmful substances over considerable distances. According to the data of the Institute of Physical Materials Science SB RAS, the probability of temperature inversions in the lower 100-m layer of the atmosphere is 77%. Under such conditions, emissions from industrial facilities and autonomous heat sources are poorly dispersed, creating high concentrations of harmful substances in the surface layer of the atmosphere in the city limits. 

Analysis of climatic data for Ulan-Ude, provided by the Buryat CHEM, and quantitative assessment of various factors indicate the predominance of processes that hinder atmospheric purification. In general, the meteorological potential of self-purification of the atmosphere in Ulan-Ude is low. It requires effective measures to limit emissions of pollutants into the atmosphere.

This study examined three groups of stationary sources of emissions of harmful substances into the air of Ulan-Ude during the heating season of 2020/2021: (1) large heating networks (CHPP-1, CHPP-2; large, centralized boilers of the Ulan-Ude energy complex, which are part of PAO “Territorial Generating Company No 14”); (2) autonomous sources (enterprises and small businesses); and (3) individual households. The total emissions from these sources during the period amounted to 83.8 thousand tons of pollutants (Table 3).

As can be seen from Table 3, individual households make the greatest contribution to the overall air pollution. In Ulan-Ude and its suburbs, there are 207 settlements and neighborhoods with 77,607 households, 77.7% of which use wood-burning stoves, and 22.3% use boilers. The total pollutant emissions from households in Ulan-Ude and its suburbs (by administrative boundaries) during the heating season 2020/2021 are shown in Table 4.

**Figure 2:**
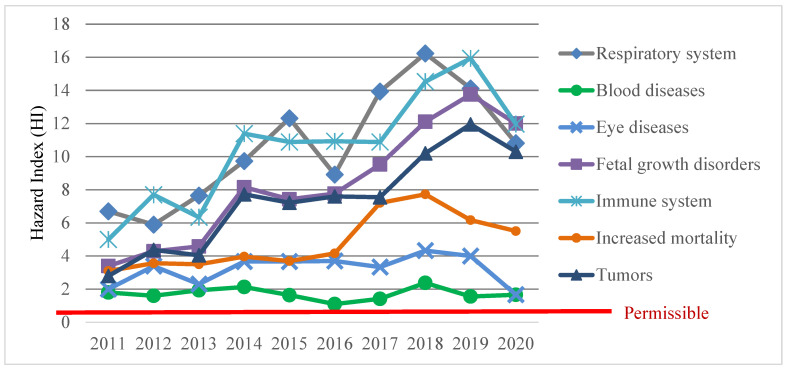
Hazard indices (HI) of non-carcinogenic risk to public health in Ulan-Ude with unidirectional effects of atmospheric air pollutants on human organs and systems in 2011–2020.

The experts of Rospotrebnadzor in Buryatia assessed the risk to public health on the basis of data on the average annual concentrations of air pollutants measured at monitoring stations. In 2020, chronic inhalation exposure to pollutants could cause disease in the population of Ulan-Ude: respiratory system (hazard index HI = 10.8, with the permissible value of 1), blood diseases (HI = 1.7), vision diseases (HI = 1.67), fetal growth disorders (HI = 12.0), immune system (HI = 11.9), increased mortality (HI = 5.5), and tumors (HI = 10.3) (Figure 2).

The hazard indices of non-carcinogenic risks to the health of the population of Ulan-Ude exceeded the permissible levels due to the content of pollutants in the air such as benzo(*a*)pyrene (HQ = 10.3), formaldehyde (HQ = 1.7), suspended solids (HQ = 1.7), PM_10_ (HQ = 1.5), PM_2.5_ (HQ = 1.8), and nitrogen dioxide (HQ = 1.1).

**Figure 3:**
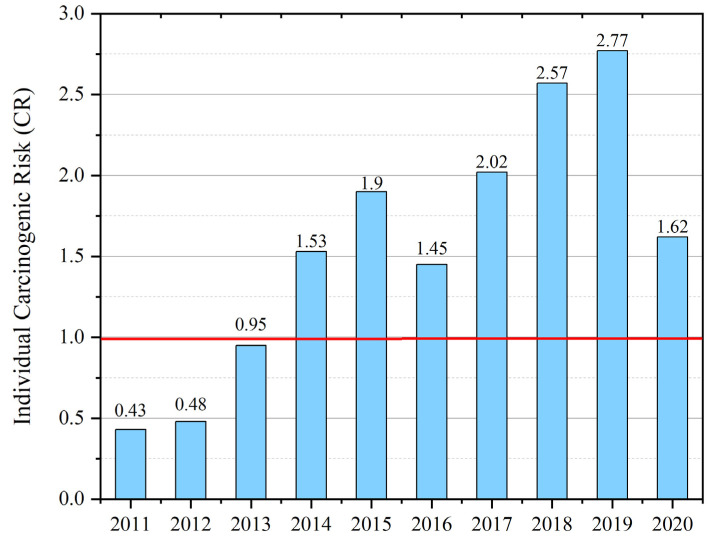
The level of individual carcinogenic risk to public health from exposure to pollutants in the atmospheric air of Ulan-Ude, 2011–2020 (CR × 10^−4^). Permissible level CR = 1 × 10^−4^.

The level of individual carcinogenic risk for the population of Ulan-Ude was 1.62 × 10^−4^, which is considered “acceptable for professional groups and unacceptable for the population”. Priority pollutants in the atmospheric air that formed the carcinogenic risk were black carbon (52.1%, CR = 0.84 × 10^−4^) and formaldehyde (40.7%, CR = 0.66 × 10^−4^) (Figure 3).

Statistics for the period 2011–2020 show that for some risk-related diseases, the morbidity of the population in Ulan-Ude exceeds the average figures for the Republic of Buryatia. The total morbidity rate in Ulan-Ude was 1.2 times higher than the average in Buryatia and amounted to 77,077.63 cases per 100 thousand people (63,985.43 cases per 100 thousand people in Buryatia). Respiratory system morbidity in Ulan-Ude was 34,154.29 cases per 100 thousand people, which is 1.19 times higher than the average for Buryatia (28,648.46 per 100 thousand people). Corresponding exceedances were registered for the diseases of endocrine system by 1.25 times, circulatory system by 1.11 times, eye diseases by 1.06 times, neoplasms by 1.47 times, and congenital anomalies, deformities, and chromosomal abnormalities by 1.63 times (Table 5).

**Figure 4:**
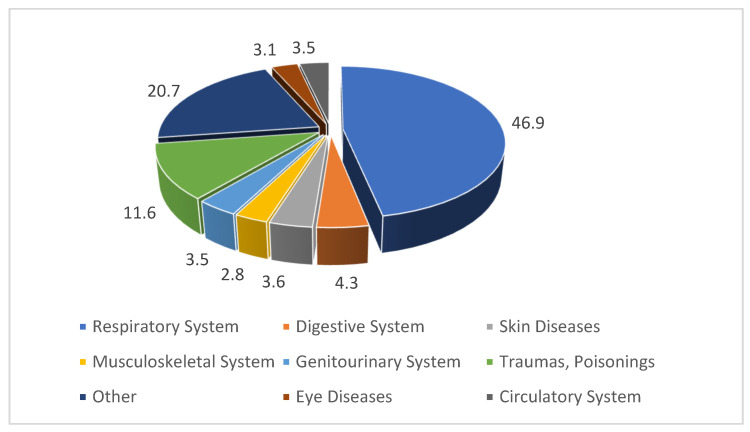
Structure of morbidity in Ulan-Ude in 2020, %.

According to Rospotrebnadzor in Buryatia, in 2020 the morbidity structure was as follows: respiratory diseases—46.9%; injuries, poisonings, and accidents—11.6%; digestive diseases—4.3%; skin diseases—3.6%; and diseases of the urogenital system and circulatory system—3.5% each (Figure 4) [45].

Respiratory diseases account for the largest share of the total morbidity rate in Ulan-Ude and Buryatia, both for 2020 and over a multi-year period. The morbidity of the population of Ulan-Ude during the period from 2011 to 2020 has increased for respiratory diseases by 11.5%, and by 8.35% for diseases of the circulatory system (Table 6).

To establish the correlation between air pollution and the growth of morbidity in Ulan-Ude, a correlation analysis was carried out with a small number of observations (n=10 years). To assess the strength of the correlation, we used the generally accepted criteria: correlation coefficient r_xy_ < 0.3 indicates a weak correlation, 0.3 ≤ r_xy_ < 0.7—average correlation, and 0.7 ≤ r_xy_—very high correlation. The correlation reliability criterion was calculated as follows: t_r_ = r_xy_/m_r_, where m_r_ is the mean error. A correlation is considered reliable if t_r_ ≥ 3.

The correlation coefficient between the atmospheric air pollution index (API_5_) and the incidence of respiratory diseases in Ulan-Ude is r_xy_ = 0.7784 (m_r_ = 0.2219, t_r_ = 3.5068, n = 10), indicating that the two data sets are strongly correlated. The correlation coefficient between API_5_ and the incidence of circulatory system diseases is r_xy_ = 0.7437 (m_r_ = 0.2363, t_r_ = 3.1471, n = 10), which also indicates a strong correlation between the two data series.

## 4. Discussion

It has been established that the degree of atmospheric air pollution in Ulan-Ude is estimated as very high. Over the past decade, the atmospheric pollution index has increased by 3.71 times due to high concentrations of benzo(*a*)pyrene, PM_2.5_, PM_10_, and nitrogen dioxide [45,46,47].

The high level of atmospheric pollution is caused by emissions from CHPPs that operate year-round, as well as emissions from autonomous heat sources of individual households during the heating period. The growing number of households with autonomous heating, the increasing amount of coal burned, and the lack of opportunities to use alternative sources of thermal energy, combined with the low potential for dispersion of harmful impurities in the atmosphere leads to increased air pollution in Ulan-Ude, both in its central part and in the suburbs.

The assessment showed that atmospheric air pollution in Ulan-Ude poses an increased risk to public health. Concentrations of pollutants in the atmospheric air have been found to present elevated levels of non-carcinogenic risk to public health, exceeding permissible values from 1.1 to 12 times. Chronic inhalation exposure to pollutants may cause health disorders of the population of Ulan-Ude from the respiratory organs, immune system, disorders of fetal development, neoplasms, diseases of the vision, blood diseases, and increased mortality. The level of individual carcinogenic risk exceeds the permissible level for the population of Ulan-Ude by 1.62 times and is estimated as “acceptable for professional groups and unacceptable for the population as a whole”. 

Analysis of the morbidity in the Ulan-Ude population revealed an increase in the incidence of risk-related diseases such as the respiratory organs and circulatory system. Their strong correlation with atmospheric air pollution in Ulan-Ude was established.

Thus, this study analyzed the data on emissions of pollutants into the atmospheric air from stationary sources, statistics on the morbidity of the population over the past decade, and the results of measurements of air quality (both at the monitoring stations and monitoring sites in micro-districts of Ulan-Ude). The results of the study confirmed that the morbidity of the population in Ulan-Ude has been increasing, and this is largely due to very high pollution of the atmospheric air.

In order to reduce carcinogenic and non-carcinogenic risks to public health in Ulan-Ude, the Directorate of Rospotrebnadzor in Buryatia has established cooperation with executive bodies and local authorities. In 2020, amendments were made to the regulatory and legal acts of the Republic of Buryatia to prohibit the use of autonomous sources of pollutant emissions into the atmosphere when there is a technical possibility of connecting to centralized heating networks. As a result of significant efforts made by the executive and legislative branches of the Republic of Buryatia, the city of Ulan-Ude has been included in the list of settlements in which the experimental quoting of pollutant emissions to the atmospheric air is carried out based on the integrated calculations of atmospheric air pollution (according to the Russian Federation Government Decree of 7 July 2022 No. 1852-r). The Directorate of Rospotrebnadzor in Buryatia initiated the updating of the summary calculations of atmospheric air pollution in Ulan-Ude, on the basis of which management decisions will be made in the framework of the federal project “Clean Air”.

## 5. Conclusions

1. In Ulan-Ude, there has been a 3.71-fold increase in air pollution over the period of 2011–2020. In 2011–2012, the degree of air pollution was assessed as “High”, and from 2013 to 2020—as “Very High”. Priority pollutants whose concentrations exceed MAC are benzo(a)pyrene, PM_2.5_, PM_10_, suspended solids, and nitrogen dioxide.
2. The main stationary sources of atmospheric air pollution are large enterprises of the fuel and energy complex, autonomous heat supply sources of small enterprises, and individual households (which make the greatest contribution to the air pollution). There has been an increase in the number of households with autonomous sources of heating.
3. Chronic inhalation exposure to pollutants may cause health disorders of the population of Ulan-Ude from the respiratory organs, immune system, disorders of fetal development, neoplasms, diseases of the vision, blood diseases, and increased mortality. The concentrations of pollutants in the atmospheric air have been found to present elevated levels of non-carcinogenic risk to public health, exceeding permissible values from 1.1 to 12 times. The level of individual carcinogenic risk exceeds the permissible level for the population of Ulan-Ude by 1.62 times. Priority pollutants in the atmosphere of Ulan-Ude whose concentrations create unacceptable levels of risk to public health are benzo(a)pyrene, suspended solids, nitrogen dioxide, PM_2.5_, PM_10_, formaldehyde, and black carbon.
4. The levels of morbidity in Ulan-Ude were higher than the average for Buryatia by the main disease classes: respiratory organs by 1.19 times, endocrine system by 1.25 times, circulatory system by 1.11 times, eye diseases by 1.06 times, neoplasms by 1.47 times, congenital anomalies, and deformations and chromosomal aberrations by 1.63 times. There is an increase in the incidence of risk-related diseases of the respiratory organs and circulatory system. A strong correlation was found between this growth of morbidity and atmospheric air pollution in Ulan-Ude.

## Figures and Tables

**Table 1:** Locations of the air monitoring stations/sites in Ulan-Ude.

No	Operator	Type	Location
1	Buryat Center for Hydrometeorology and Environmental Monitoring	Monitoring stations	Prospekt 50-letiya Oktyabrya (ASK-A No.1)
2			Ulitsa Revolutsii 1905 (ASK-A No.6)
3			Ulitsa Babushkina, section No.16 (ASK-A No.2)
4	Rospotrebnadzor	Monitoring sites	Ulitsa Sovetskaya, 43; near school No.3 (Site 1)
5			Ulitsa Mokhovaya, 1; near kindergarten “Pchelka”, influence zone of CHPP-1 (Site 2)
6			Ulitsa Rodiny, 2; square in the influence zone of the boiler house of Zagorsk (Site 3)
7			Prospekt Stroitelei, 20; near school No. 49 (Site 4)
8			Ulitsa Klyuchevskaya, 45B; near the Head Office of Rospotrebnadzor in Buryatia (Site 5)
9			Ulitsa Zabaikalskaya, 2, Silikatny; influence zone of the settlement’s enterprises (Site 6)
10			Ulitsa Stroitelei, 19A, Zarechny; near kindergarten “Zorka” (Site 7)

**Table 2:** Dynamics of the average annual concentrations of pollutants, API, and degree of air pollution in Ulan-Ude in 2011–2020 (according to the Buryat CHEM), in shares of MAC.

Pollutant	Years										Change by 2020 %	Note
	2011	2012	2013	2014	2015	2016	2017	2018	2019	2020		
Nitrogen dioxide	1	1.10	1.10	1.13	1.05	1	0.93	1	0.95	1.08	7.5	vs. 2011 year
Suspended matter	1.5	1.7	1.7	1.9	1.76	1.77	1.75	1.88	1.48	1.30	−13.3	
Carbon monoxide	0.5	0.50	0.50	0.43	0.20	0.13	0.17	0.17	0.19	0.17	−66.7	
Sulfur dioxide	0.1	0.10	0.10	0.17	0.18	0.2	0.26	0.36	0.29	0.20	100.0	
Formaldehyde	2	2.30	2.30	1.78	1.10	1.1	1.00	1.30	1.2	0.50	−75.0	
Phenol	0.8	0.90	0.90	0.80	0.66	1	1	1	0.56	1.00	25.0	
Benzo(*a*)pyrene	2.8	2.8	4	7.7	7.22	6.8	7.6	10.2	11.95	10.30	367.9	
Nitrogen oxide	0.2	0.20	0.20	0.56	0.36	0.23	0.32	0.42	0.41	0.20	0	
Ozone	−	−	−	−	1.53	1.23	0.9	1.07	1.17	0.73	−52.1	vs. 2015 year
Ammonia	−	−	−	−	0.20	0.35	0.2	0.10	0.02	0.02	−90.0	vs. 2015 year
Black carbon	−	−	−	−	1.04	0.86	0.28	0.34	0.48	0.38	−63.5	vs. 2015 year
PM_10_	−	−	−	−	−	−	1.2	1.13	1.07	1.50	25.0	vs. 2017 year
PM_2.5_	−	−	−	−	−	−	1.34	1.37	1.34	1.76	31.3	vs. 2017 year
API_5_	10.0	12.4	14.6	27.3	25.2	22.9	25.6	38.0	46.3	37.1	371	vs. 2011 year
Degree of air pollution	High	High	Very high	Very high	Very high	Very high	Very high	Very high	Very high	Very high		

‘−’—data not available.

**Table 3:** Air emissions of pollutants from stationary sources in Ulan-Ude during the heating season of 2020/2021 (thousand tons).

No.	Type of Stationary Source	Mass (Thousand Tons)	wt.%
1	Large heating networks (fuel and energy complex)	18.0	21.5
2	Autonomous sources (enterprises and small businesses)	2.0	2.4
3	Individual households	63.8	76.1
4	Total	83.8	100

**Table 4:** Total pollutant emissions from households in Ulan-Ude and its suburbs per year (during the heating season 2020/2021.

Area	Pollutant					
	Benzo(*a*)pyrene	Nitrogen Oxides (NO_X_)	Sulfur Dioxide (SO_2_)	Particulate Matter	Carbon Monoxide (CO)	Total
	kg	Thousand Tons				
Ulan-Ude	10.29	0.22	7.33	25.87	5.85	39.27
Ulan-Ude suburb, located in Tarbagataisky District	1.04	0.02	0.74	2.61	0.59	3.96
Ulan-Ude suburb, located in Ivolginsky District	3.53	0.07	2.52	8.88	2.01	13.48
Ulan-Ude suburb, located in Zaigraevsky District	1.84	0.04	1.31	4.63	1.05	7.03
Total	16.7	0.36	11.9	41.99	9.5	63.75

**Table 5:** Comparative characteristics of morbidity in Ulan-Ude and Buryatia for 2011–2020.

Disease Classes	Ulan-Ude		Buryatia		Excess Rate (Ulan-Ude vs. Buryatia
	Cases, per 100,000 People	% of Total Morbidity	Cases, per 100,000 People	% of Total Morbidity	
Respiratory organs	34,154.29	44.31	28,648.46	44.77	1.19
Congenital anomalies, deformities, and chromosomal abnormalities	151.53	0.20	92.74	0.14	1.63
Diseases of the eye	3149.95	4.09	2977.82	4.65	1.06
Circulatory system	2837.52	3.68	2551.25	3.99	1.11
Blood and hematopoietic organs	383.85	0.50	462.3	0.72	0.83
Neoplasms	1073.08	1.39	731.89	1.14	1.47
Endocrine system	1673.95	2.17	1338.39	2.09	1.25
Others	33,653.46	43.66	27,182.58	42.5	1.24
Total	77,077.63	100.00	63,985.43	100.00	1.20

**Table 6:** Dynamics of population morbidity in Ulan-Ude for 2011–2020 (cases per 100,000 population).

Disease Classes	Year									
	2011	2012	2013	2014	2015	2016	2017	2018	2019	2020
Respiratory diseases	32,359.0	33,397.4	34,231.0	34,771.6	32,409.9	33,925.6	33,569.7	34,956.3	35,842.1	36,080.3
Congenital anomalies, deformities, and chromosomal abnormalities	206.7	193.1	211.0	217.3	89.5	120.2	124.7	120.9	116.6	115.3
Diseases of the eye	4127.7	3529.2	3116	3937.1	3445.2	2735.6	2880.8	2638.3	2725.4	2364.2
Circulatory system	2491.7	2351.8	2549.8	2560.7	2669.9	3140.1	3174.1	3221.6	3515.8	2699.7
Blood and hematopoietic organs	338.3	387.5	405.5	389.8	391.0	408.6	436.8	424.1	396.9	260.0
Neoplasms	1085.8	1197.1	1114.0	1219.1	1058.6	1073.7	1037.8	969.3	1078.5	896.9
Endocrine system	1756.7	1687.9	1661.5	1854.1	1620.7	1727.5	1838.5	1456.4	1803.9	1332.3
Others	38,095.5	38,209.50	34,916.3	38,096.3	29,526.0	30,417.6	31,912.1	31,065.2	31,038.7	33,257.4
Total morbidity	80,461.4	80,953.5	78,205.1	83,046.0	71,210.8	73,548.9	74,974.5	74,852.1	76,517.9	77,006.1

## Data Availability

Not applicable.
